# Dr. Edward Lawson

**Published:** 1870-10

**Authors:** 


Dr. Edward Lawson (*Practitioner*) says, the sulpho-carbolates are not powerful *direct* antiseptics; they are far less powerful than carbolic acid; half a grain of pure acid is equal to twenty grains of sulpho-carbolate of sodium. Not only is the salt as compared with the acid far less efficient in destroying the life of vegetable cells, but the acid loses in this power by combination with the base; *i. e.*, one grain of the acid, when free, is as efficient as twenty times that amount in combination. But these salts can be readily administered without difficulty or danger; *in transitu* throughout the system the carbolic acid becomes evolved, producing its effect. In the sulpho-carbolate we are enabled in effect to administer carbolic acid in an innocuous form, and in larger quantity than otherwise possible.

The *sulpho-carbolate of zinc* has been used as an external application in the form of lotion. Ten grains should be dissolved in an ounce of distilled water. It abolishes the fetor of pus-forming surfaces, and is superior to carbolic acid on account of the absence of irritating qualities, and of all unpleasant odor.

For internal use, the sulpho-carbolate of sodium is preferable; its solution in water has no unpleasant taste. The dose is 20 to 60 grains.

Given in phthisis, while it has no power to arrest the disease, there was a steady gain in nutrition; in many cases the strength increased, rest improved, the fetor of expectoration was arrested, the night sweats decreased, and the appetite improved.

I employed it in five cases of severe tonsillar ulceration, which all rapidly recovered without suppuration.—*Half-Yearly Compend.*

